# Incorporation of nanogels within calcite single crystals for the storage, protection and controlled release of active compounds†

**DOI:** 10.1039/d1sc02991f

**Published:** 2021-06-28

**Authors:** Ouassef Nahi, Alexander N. Kulak, Thomas Kress, Yi-Yeoun Kim, Ola G. Grendal, Melinda J. Duer, Olivier J. Cayre, Fiona C. Meldrum

**Affiliations:** School of Chemistry, University of Leeds Woodhouse Lane Leeds LS2 9JT UK pmona@leeds.ac.uk f.meldrum@leeds.ac.uk; School of Chemical and Process Engineering, University of Leeds Woodhouse Lane Leeds LS2 9JT UK; Yusuf Hamied Department of Chemistry, University of Cambridge Lensfield Rd. Cambridge CB2 1EW UK; The European Synchrotron Radiation Facility (ESRF) 71 Avenue des Martyrs 38000 Grenoble France

## Abstract

Nanocarriers have tremendous potential for the encapsulation, storage and delivery of active compounds. However, current formulations often employ open structures that achieve efficient loading of active agents, but that suffer undesired leakage and instability of the payloads over time. Here, a straightforward strategy that overcomes these issues is presented, in which protein nanogels are encapsulated within single crystals of calcite (CaCO_3_). Demonstrating our approach with bovine serum albumin (BSA) nanogels loaded with (bio)active compounds, including doxorubicin (a chemotherapeutic drug) and lysozyme (an antibacterial enzyme), we show that these nanogels can be occluded within calcite host crystals at levels of up to 45 vol%. Encapsulated within the dense mineral, the active compounds are stable against harsh conditions such as high temperature and pH, and controlled release can be triggered by a simple reduction of the pH. Comparisons with analogous systems – amorphous calcium carbonate, mesoporous vaterite (CaCO_3_) polycrystals, and calcite crystals containing polymer vesicles – demonstrate the superior encapsulation performance of the nanogel/calcite system. This opens the door to encapsulating a broad range of existing nanocarrier systems within single crystal hosts for the efficient storage, transport and controlled release of various active guest species.

## Abstract 2

Nanocarriers have tremendous potential for the encapsulation, storage and delivery of active compounds.

## Graphical abstract

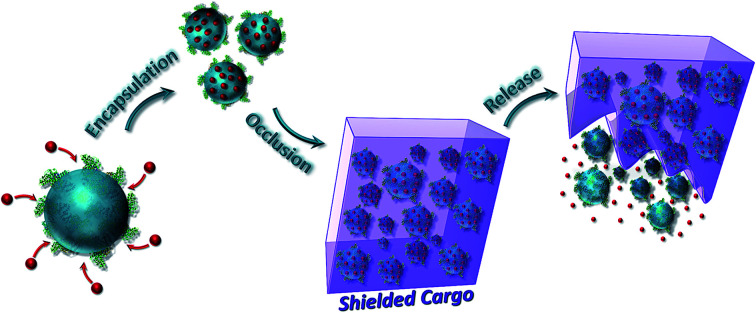

## Introduction

The creation of effective carrier systems that can stabilize active compounds for extended periods before offering controlled release is crucial for many biomedical, agricultural, food, personal care and cosmetic applications.^1–3^ These challenges have been addressed by encapsulating active species such as drugs, proteins, enzymes and RNA/DNA^1,4^ within a range of carriers. Of these, organic nanoparticles including liposomes, dendrimers and polymer nano-objects have been widely explored due to their biocompatibility, biodegradability and the ability to tune their structures and chemistry.^1,4–7^ Nanogels, in particular, have garnered considerable attention due to their unique combination of properties including tunable sizes, high-water contents and distinct swelling behaviors.^7–9^ Further, high loading efficiencies can be achieved and release kinetics can be tuned according to the molecular architecture of the nanogels.^7^ However, as stand-alone systems, instability and uncontrolled leaching of the payloads limits their efficacy.^7,10^

To overcome the problems associated with organic carriers, active compounds have been incorporated within biocompatible inorganic hosts such as silica, calcium carbonate and calcium phosphate.^11–13^ This is usually accomplished by locating the active agents within mesoporous, amorphous or polycrystalline particles, but all suffer problems with leakage associated with an inherent porosity or physical instability (amorphous carriers). Even more elaborate approaches including forming Pickering emulsions^14,15^ or creating mineral shells^16,17^ seldom succeed in eliminating all leakage.^18–20^ The encapsulation of active agents within impermeable metal shell capsules has proven effective in this regard,^21–23^ but these are prepared using multi-step processes and the release of the payloads can only be initiated in a burst, by breaking the shell. The creation of a hybrid system that combines the advantages of both organic and inorganic carriers would therefore be highly attractive.

**Fig. 1:**
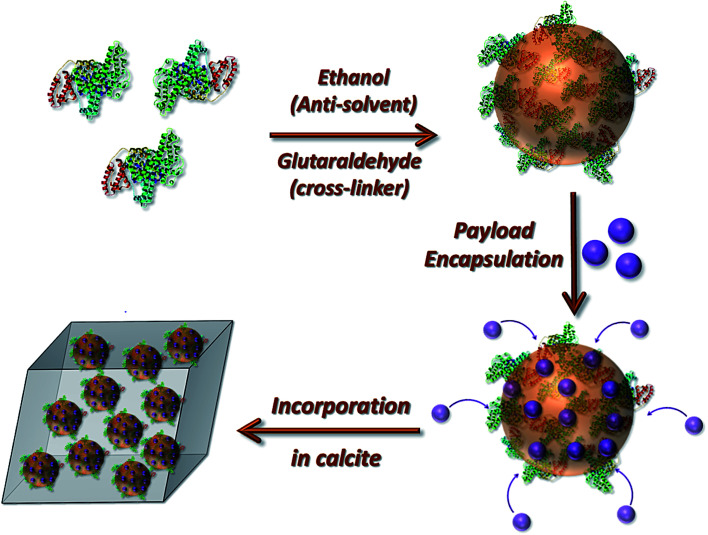
Schematic diagram illustrating the preparation of the loaded protein nanogels and their subsequent incorporation within calcite single crystals.

We here realize this goal by developing a strategy in which loaded nanogels are occluded within single crystals of calcite (CaCO_3_), leading to their protection within an impermeable inorganic host (Fig. 1). Crystal growth is a traditional method of purification, and as such co-precipitation with bioactive molecules typically leads to low levels of occlusion.^24–26^ However, we have recently shown that it is possible to achieve high loadings of inorganic nanoparticles^27–29^ and polymer nano-objects^30–32^ provided that the surfaces are functionalized with polymer chains or proteins such that the particles are both colloidally stable in the crystal growth solution and bind to the crystal surface. Notably, polymer vesicles containing small fluorescent dyes^33^ and silica particles^33,34^ have been occluded, but the highly permeable vesicle membrane precludes their general use as effective carriers.^6,33,35,36^ The potential of this system for protecting and delivering active compounds has therefore never been explored.

The work described here shows that bovine serum albumin (BSA) nanogels impregnated with active species including doxorubicin (DOX, a widely used chemotherapeutic drug)^37,38^ and lysozyme (an antibacterial model enzyme)^39^ can be effectively occluded throughout calcite single crystals using a simple one-pot method. Notably, this is achieved with as-synthesized nanogels, such that no additional synthesis of custom-made polymers or functionalization of the nanogels is required. The mineral is shown to effectively protect the encapsulated active compounds from harsh external conditions and controlled release can be initiated by simply changing the pH of the medium to dissolve the host crystal. This system is therefore both simple and versatile, and effectively combines the established properties of nanogels with those of the impermeable mineral host. The advantages of this single crystal-based system are also further demonstrated by comparison with amorphous calcium carbonate (ACC) and polycrystalline vaterite as carrier materials, and loaded polymer vesicles as the encapsulated species.

## Results

### Synthesis of bovine serum albumin (BSA) nanogels

**Fig. 2:**
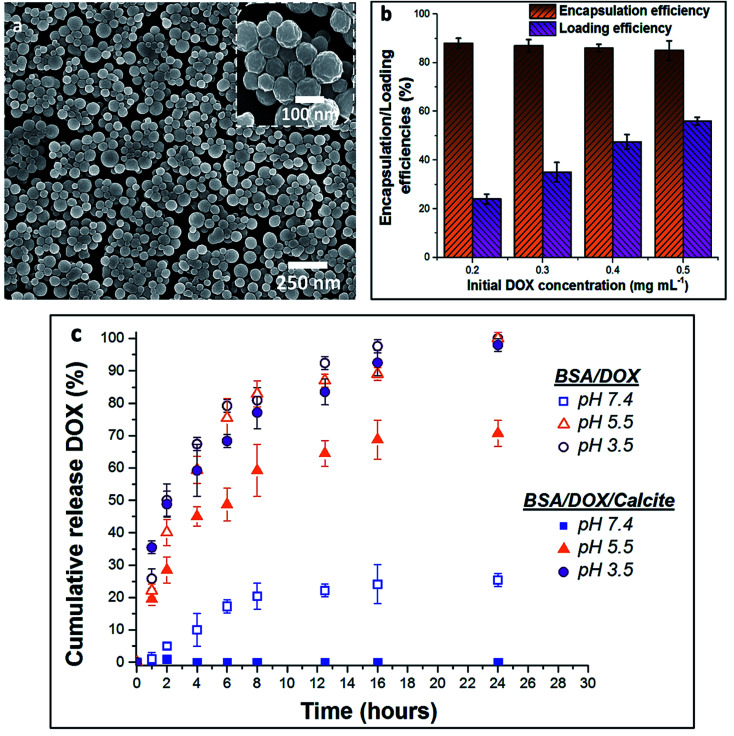
(a) SEM micrograph of the dry BSA nanogels. (b) Chart of the encapsulation and loading efficiencies of DOX within the BSA nanogels, depending on the initial drug feed. (c) Release profiles of DOX–BSA nanogels (open symbols) and DOX–BSA nanogels occluded within calcite (full symbols) at physiological pH = 7.4 (PBS buffer; 37 °C) and endosomal/lysosomal pH = 3.5 and 5.5 (citrate buffers; 37 °C). The release kinetics show that occlusion of the loaded nanogels within calcite prevents undesired leakage at pH 7.4 and enables controlled release of the drug in solution at lower pH. The error bars represent the standard deviations for 3 consecutive measurements.

BSA nanogels were prepared using a facile desolvation method in which ethanol was added to an aqueous solution containing BSA molecules.^40^ A small amount of glutaraldehyde (final concentration 10^−3^ wt%) was then added to chemically cross-link and stabilize the nanoparticles. Dynamic Light Scattering (DLS) of the nanogels suspended in water showed that they have a narrow size distribution (polydispersity index (PDI) = 0.05; standard deviation (*σ*) = 55 nm) and a mean hydrodynamic size of 250 nm (Fig. S1†), while Scanning Electron Microscopy (SEM) of dry nanogels revealed spherical nanoparticles with diameters of 100–150 nm (Fig. 2a). This difference in size highlights the high-water content of the gels.^9^

### Encapsulation and release of DOX from BSA nanogels

The encapsulation and loading efficiencies of DOX in the BSA nanogels were assessed, *via* the following equations:1
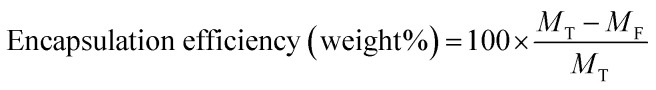
2
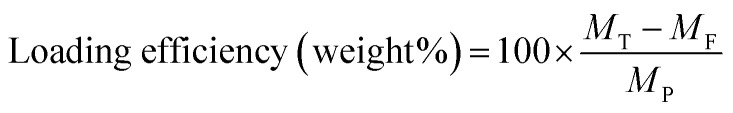
where *M*_T_ is the total mass of DOX fed into the nanogels suspension, *M*_F_ is the mass of free DOX that is not encapsulated within the nanogels and *M*_P_ is the mass of the BSA nanogels added in the suspension. Very high loading efficiencies of ≈24–55% were achieved for drug feeds between 0.2 mg mL^−1^ and 0.5 mg mL^−1^, and high encapsulation efficiencies of ≈87% were obtained across the whole range of initial DOX concentrations investigated (Fig. 2b). DOX feeds of 0.5 mg mL^−1^ offered the optimum encapsulation performance and were employed in subsequent experiments.

In turn, DOX release was investigated by immersing loaded nanogels in buffered solutions with different pH values and monitoring the amount of DOX released using UV-visible spectrophotometry (Fig. 2c). 20% was released within 8 h and up to 25% after 24 h of incubation at a physiological pH of 7.4 (PBS buffer; 37 °C). This compares with 45% within 2 h, and 80% after 8 h at a lower endosomal/lysosomal pH of 5.5 or 3.5 (citrate buffers; 37 °C). These differences are due to the pH-dependent binding affinity of DOX with BSA.^37,38^ DOX is always positively charged under the pH conditions explored in this study,^37^ while BSA has an isoelectric point (IEP) around pH ≈5 and is negatively charged at pH 7.4.^41^ The electrostatic interactions between BSA and DOX therefore change from attractive to repulsive as the pH is reduced, leading to an accelerated release of DOX. Importantly, although high loading efficiencies can be achieved at pH 7.4, DOX still leaches from the nanogels over time when it is not incorporated within calcite single crystals, limiting practical applications.^37,38^

### Incorporation of nanogels in calcite single crystals

**Fig. 3:**
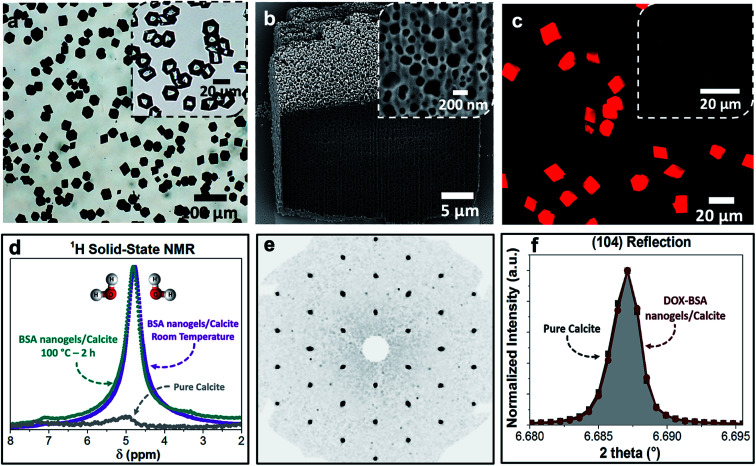
(a) Optical image of the BSA nanogels occluded in calcite single crystals. The inset shows calcite crystals precipitated in the presence of DOX only. The colorless and transparent crystals indicate that DOX molecules do not incorporate in calcite on their own. (b) FIB-SEM micrograph showing the uniform incorporation of the DOX–BSA nanogels in calcite. (c) Fluorescence image of the DOX–BSA nanogels occluded in calcite (under UV irradiation at 365 nm). Inset is the non-fluorescent BSA nanogel/calcite composites under UV light. The highly fluorescent DOX-nanogel/calcite composites provide evidence of the efficient incorporation of the drug within the hybrid crystals. (d) ^1^H solid-state NMR of the composites revealing that they are highly hydrated, even after annealing at 100 °C for 2 h. (e) Single-crystal XRD of the hybrid showing that incorporation of the nanogels does not alter the single crystallinity of the calcite host. (f) HR-PXRD showing the main (104) reflection of pure calcite and DOX–BSA nanogels incorporated in calcite.

Loaded nanogels were then occluded within calcite single crystals to overcome the limitations of the nanogels as a stand-alone carrier system. Hybrid crystals were synthesized by adding the DOX-loaded BSA nanogels to a CaCl_2_ solution and initiating crystallization by exposure to ammonium carbonate vapors.^42^ Well-defined rhombohedral calcite crystals formed (Fig. 3), and the polymorph was confirmed using Raman spectroscopy (Fig. S2†) and powder X-ray diffraction (*p*-XRD) (Fig. S3†). Thermogravimetric analysis (TGA) (Fig. S4a†) demonstrated a high incorporation efficiency of up to 45 vol%, which is consistent with the dark color of the composite crystals (Fig. 3a) and the observation of encapsulated protein using Fourier Transform Infrared (FTIR) (Fig. S5†). Scanning electron imaging of cross-sections through the crystals confirmed that the nanogels were uniformly incorporated throughout the calcite host (Fig. 3b).

Further characterization was also conducted to investigate the effect of occlusion on the nanogels and the entrapped DOX, and on the calcite host itself. ^1^H solid-state NMR analysis of the composite crystals revealed an intense peak at *δ* ≈ 4.9 ppm that corresponds to water molecules (Fig. 3d), which is not present in pure calcite crystals. This confirms that the nanogels are incorporated in the crystals in their native, hydrated state, such that they offer an ideal environment for the storage of active compounds.^1,5–7,10^ The characteristic fluorescence of DOX within the crystals showed that the drug was retained within the nanogels during incorporation in calcite (Fig. 3c). Single-crystal XRD of the hybrid showed that incorporation of the nanogels did not affect the single crystallinity of the calcite host (Fig. 3e). High-resolution synchrotron p-XRD analysis of the individual diffraction peaks (the (104) is shown in Fig. 3f) revealed no change in the peak position or shape, and thus demonstrates that even at these high loadings, the nanogels do not alter the microstructure of the host calcite crystals.^43^

### Release of DOX from nanogel-calcite crystals

The release profile of DOX from the host crystals was then evaluated by incubating the hybrid crystals in buffered solutions of different pH values, and monitoring DOX release over time. Importantly, no release of DOX could be detected at pH 7.4 after 24 h of incubation, as compared to 25% release in the absence of the mineral within the same period (Fig. 2c). In contrast, 29% DOX was released after 2 h at pH 5.5, 59% after 8 h and 71% after 24 h. This compares to 40% of DOX released after 2 h in the absence of the calcite host. Yet faster release occurred at pH 3.5, where 49% of DOX was discharged within 2 h, 80% after 8 h and 100% after 24 h. This release profile derives from the solubility of calcium carbonate in acidic media,^44^ combined with electrostatic repulsions between DOX and the BSA nanogels at acidic pH, which also facilitate release.

### Encapsulation and protection of lysozyme in BSA nanogel-calcite crystals

**Fig. 4:**
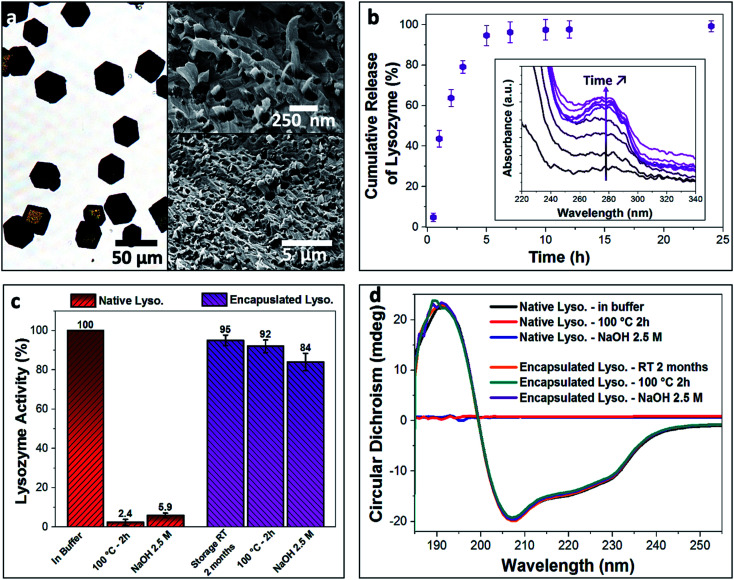
(a) Optical image of the lysozyme-loaded BSA nanogel/calcite system and SEM micrographs of a fractured composite crystal showing the uniform occlusion of the loaded nanogels in calcite. (b) Cumulative release of lysozyme from the composite crystals over time (citrate buffer, pH = 3.5). Inset shows the increased absorbance of the characteristic peak of lysozyme (*λ* = 280 nm) corresponding to the released enzyme over time. (c) Native (red) and encapsulated (purple) lysozyme activity stored at room temperature for 2 months and subjected to heat treatment at 100 °C for 2 h or NaOH solution (2.5 M, pH 14). Encapsulation of lysozyme within the BSA/calcite system enables preservation of enzymatic activity compared to the free enzyme in solution. (d) CD spectra of the native and encapsulated lysozyme when stored at room temperature for 2 months and heated at 100 °C for 2 h or subjected to high basic solution (2.5 M NaOH, pH 14) for 2 h. Compared to the free native enzyme, the encapsulated lysozyme retains its secondary structure and therefore stability when exposed to extreme conditions.

Enzymes are highly sensitive to their surrounding environment and often lose their bioactivity due to aggressive external stresses.^45^ As a model system, we explored the ability of our nanogel/calcite hybrid crystals to protect lysozyme against harsh external conditions. Lysozyme was first encapsulated within the BSA nanogels with a high loading efficiency of 59%, and these were then incorporated within calcite single crystals (Fig. 4a). The hybrid crystals were subsequently exposed to (1) a 2.5 M solution of NaOH with pH 14 for 2 h, or (2) heat treatment at 100 °C for 2 h. The enzyme was then released from the host crystals in buffered solution at pH 3.5 (citrate buffer; 37 °C) (Fig. 4b), and 95% discharge of the payload was achieved within 5 h. This fast release is attributed to the electrostatic repulsions between the positively charged lysozyme (IEP at pH ≈ 11) and BSA nanogels (IEP at pH ≈ 5), accompanied by the high solubility of calcite in acidic solution. The activity of the released enzyme was assessed using *Micrococcus lysodeikticus* cells (Fig. 4c).

Once the released enzyme was recovered, our tests showed that almost full enzymatic activity (Fig. 4c, enzymatic activity ≈ 95%) was retained when lysozyme-BSA/calcite crystals were stored at room temperature (20 °C) for 2 months, or when exposed to the basic solution (Fig. 4c, enzymatic activity ≈ 84%). A high enzymatic activity of up to 92% was also retained following the heat treatment. In contrast, only 5.9% and 2.4% of activity were recorded for native lysozyme (*i.e.*, not encapsulated), when subjected to the basic solution and heat treatment, respectively. Retention of the encapsulated lysozyme activity on exposure to the highly basic solution demonstrates the impermeability of the calcite single crystals, such that they fully shield the payload. That the enzyme is also protected against heat could be attributed to the restricted conformational freedom accessible to lysozyme that is tightly bound to the BSA nanogels.^46,47^ Further, TGA analysis (Fig. S4b†) of the composites suggests that the loaded BSA nanogels are resilient against thermal degradation when occluded in calcite, where no significant weight loss was observed for the BSA nanogel/calcite hybrid at 100 °C. Conversely, a considerable weight loss (2.5 wt%) was recorded for the non-occluded BSA nanogels. ^1^H solid-state NMR analysis also confirmed that the water was retained within the BSA nanogel/calcite hybrid crystals after annealing at 100 °C for 2 h (Fig. 3e). These results therefore suggest that a higher activation energy is required for both dehydrating and degrading the loaded nanogels incorporated within calcite compared to the non-occluded nanocarriers, possibly because of the restricted mobility of molecules in the dense mineral.^47^

Potential changes in the structure of the encapsulated lysozyme when the crystals were subjected to the external stresses were further explored using Circular Dichroism (CD) in the far-UV (185–260 nm) (Fig. 4d). Untreated free lysozyme in buffered solution was used as a control sample, where the helical structure of the enzyme yields a characteristic negative peak at 222 nm, while the α-helices and β-sheet components of lysozyme generate a negative peak at 209 nm.^39^ Temperature-dependent CD spectra of native lysozyme revealed a melting temperature of 47 °C, above which the lysozyme becomes unstable and denatures (Fig. S6†). The secondary structure of the free enzyme was also completely lost on exposure to the basic solution. In contrast, no changes in the CD spectra of the encapsulated lysozyme were observed when the crystals were stored for 2 months at 20 °C or exposed to the high temperature and pH conditions. This confirms that the encapsulation strategy does not damage the payload and that the nanogel/calcite hybrid offers high levels of protection of bioactive compounds against extreme conditions, providing exciting new prospects for storing and extending the shelf-life of proteins, vaccines and many more biologically-relevant agents.

### Comparison with encapsulated polymer vesicles

**Fig. 5:**
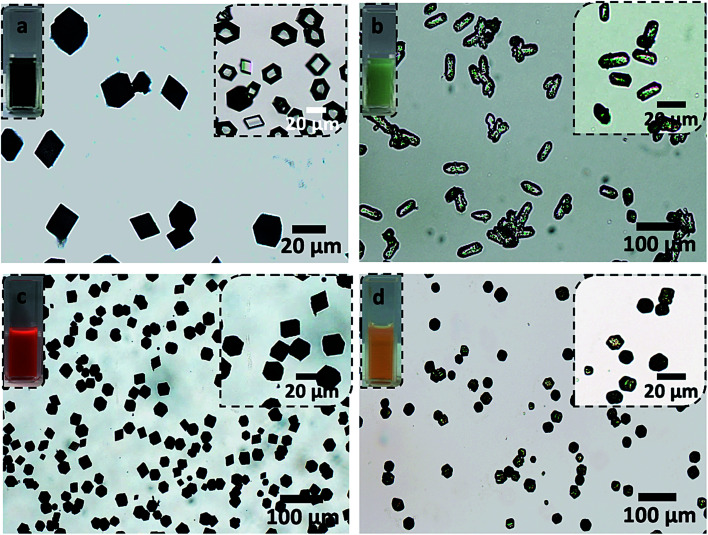
Optical micrographs of (a) IR820-BSA nanogels, (b) IR820-vesicles, (c) DOX–BSA nanogels and (d) DOX-vesicles incorporated within calcite crystals. The inset on the right in (a) shows calcite crystals precipitated in the presence of IR820 only. The transparent and colorless aspect of the crystals indicates that the IR820 molecules do not incorporate within calcite on their own. The insets on the left show the loaded nanocarriers dispersed in aqueous solutions. The darker colors of the solutions and crystals occluding the loaded BSA nanogels (a and c) compared to the vesicles (b and d) demonstrate the superior performances of the BSA nanogels as nanocarriers for both DOX and IR820, compared to the vesicles.

The performance of the BSA nanogel/calcite system was then compared with calcite crystals containing polymeric vesicles (Fig. 5), since the latter has been proposed as an effective delivery system for small fluorescent dyes,^33^ and larger payloads including silica particles^33,34^ and proteins.^35,36^ Poly(methacrylic acid)-poly(benzyl methacrylate) (PMAA-PBzMA) polymer vesicles were prepared by polymerization-induced self-assembly (PISA),^48^ since this block copolymer efficiently incorporates within calcite (Fig. S7†).^30,34^ DLS analysis of the vesicles revealed a unimodal size distribution (PDI = 0.09; *σ* = 98 nm) and a mean hydrodynamic diameter of 325 nm that is comparable to the size of the BSA nanogels (Fig. S1 and S7†).

BSA nanogels and PMAA-PBzMA vesicles were loaded with DOX or IR820 (a cyanine dye and potential candidate for near-infrared fluorescence bio-imaging).^49^ A DOX feed of 0.5 mg mL^−1^ gave an encapsulation efficiency of 35% and a loading efficiency of 20% within the vesicles, which represents a 2.5-fold decrease in performance as compared with the BSA nanogels. Similarly, a loading efficiency of only 18% was achieved in the vesicles for IR820 at a feed of 0.5 mg mL^−1^ as compared to 50% in the BSA nanogels. This could be attributed to the wider range of binding sites present on BSA compared to the vesicles, allowing the payloads to anchor more efficiently to the nanogels through electrostatic interactions, hydrogen bonding and both hydrophilic and hydrophobic interactions.^40,50^

### Comparison with DOX loaded in amorphous calcium carbonate (ACC) and vaterite

**Fig. 6:**
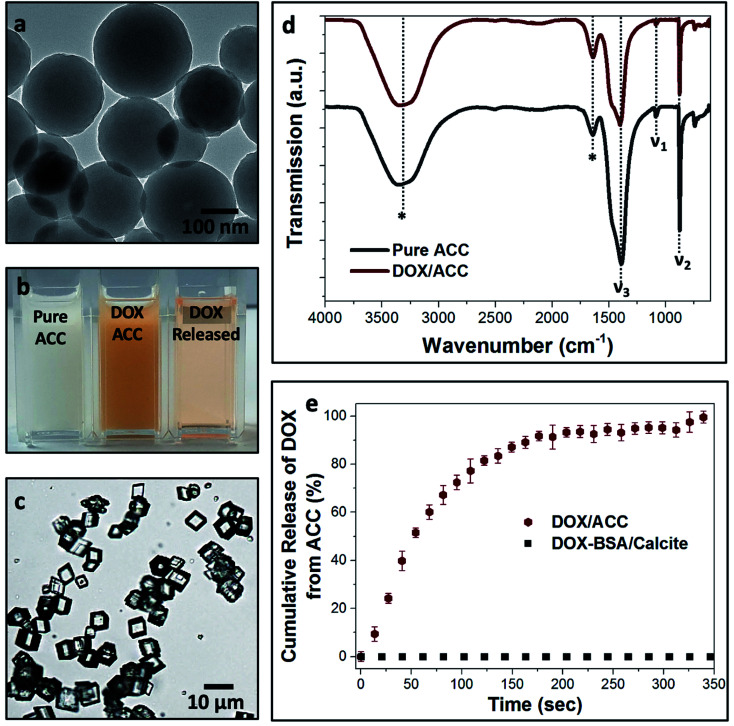
(a) Transmission electron micrograph of ACC nanoparticles co-precipitated in the presence of DOX. (b) Digital images of pure ACC, DOX/ACC and the released DOX from the ACC particles. (c) Calcite crystals precipitated at the expense of ACC dissolution. The colorless aspect of the crystals indicates that no DOX is incorporated within calcite. (d) FTIR spectra of pure ACC (grey) and DOX/ACC (red), showing the characteristic vibrational peaks of ACC: *ν*_1_ (symmetric stretching), *ν*_2_ (out-of-plane CO_3_ bending) and *ν*_3_ (splitting of the asymmetric CO_3_ stretching). The vibrational bands marked with asterisks are characteristic of water, indicative of the high hydration of the ACC nanoparticles that is responsible for their dissolution in aqueous solution. (e) Leaching of DOX from ACC over time (red), while no release of the drug is recorded for the DOX–BSA nanogel/calcite system (black).

Finally, we compared calcite single crystals as carriers with the widely used amorphous calcium carbonate (ACC) and vaterite, which are metastable forms of CaCO_3_. Precipitation of ACC in the presence of DOX yielded spherical particles with average sizes of 250 nm (Fig. 6a), and the polymorph was identified by FTIR (Fig. 6d). A DOX feed of 0.5 mg mL^−1^ yielded a loading efficiency of only 5% (Fig. 6b), in marked contrast to the 55% loading efficiency of DOX in the BSA nanogels. DOX also rapidly leached from ACC nanoparticles suspended in solution (pH 7.4; 37 °C) such that full release occurred within 5 min (Fig. 6e). This is principally due to (1) the poor binding affinity of DOX to ACC and (2) the high solubility of ACC in water (Fig. 6c). In contrast, no release of DOX was observed from the BSA nanogel/calcite system at pH 7.4.

**Fig. 7:**
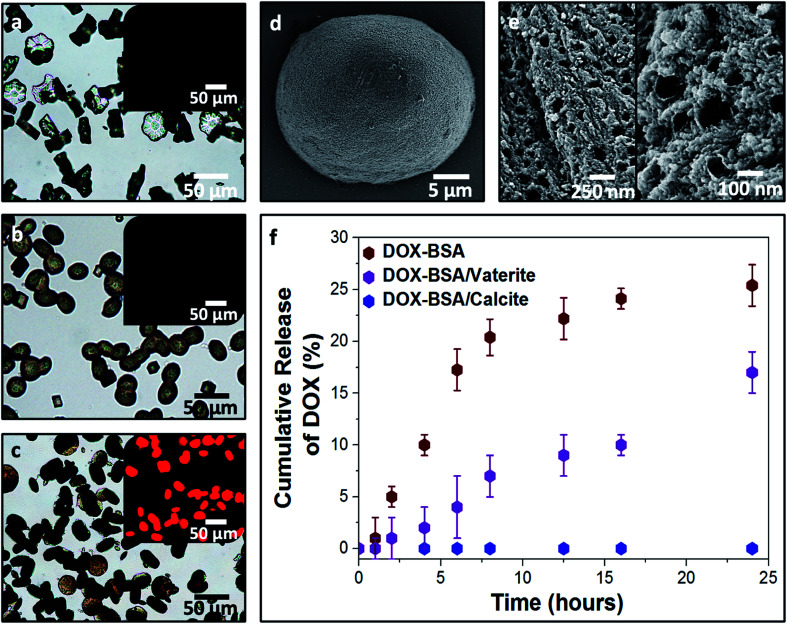
Optical images of pure vaterite (a), BSA nanogel/vaterite (b) and DOX-loaded BSA nanogel/vaterite crystals (c). Insets are the fluorescence images of the crystals under UV light (*λ* = 365 nm), showing the encapsulation of DOX within the BSA/vaterite system. SEM micrograph of a DOX–BSA nanogel/vaterite hybrid crystal (d) and cross-sections of the composites (e) revealing the loaded nanogels incorporated within the mesoporous vaterite crystals. (f) Leaching of DOX from the BSA nanogel/vaterite composites (purple) in comparison to the uncontrollable release of DOX from the BSA nanogels alone (red). Conversely, no drug leaching is recorded for the DOX–BSA nanogel/calcite system (blue).

DOX-loaded BSA nanogels were also incorporated into vaterite polycrystals (Fig. 7). Cross-sections through the crystals (Fig. 7e) and fluorescence microscopy (Fig. 7a–c) confirmed that the nanogels and DOX were incorporated, and the polymorph was determined using Raman (Fig. S2†) and FTIR analyses (Fig. S8†). Release experiments were performed at physiological pH 7.4 (PBS buffer; 37 °C) (Fig. 7f) and a gradual leaching of the drug from vaterite was recorded, reaching 17% after 24 h. This is comparable to DOX leaching from the BSA nanogels alone. No release of DOX from the BSA/calcite system was detected under the same conditions. DOX leaches from vaterite due to its polycrystalline structure (Fig. 7e), where Brunauer–Emmett–Teller (BET) analysis revealed a surface area of 20 m^2^ g^−1^ and average pore size of 10 nm (Fig. S9†). DOX molecules entrapped between the crystal domains can therefore readily leach from the vaterite particles.

## Discussion

Advances in encapsulation technologies have led to the design of numerous nanomaterials for the storage and controlled release of many active agents. Organic nanocarriers such as synthetic polymer particles (*e.g.*, nanogels, polymersomes and dendrimers)^1,4,7,10^ and bio-capsules (*e.g.*, liposomes, DNA cages and protein capsids)^6,51,52^ have been widely explored, but often suffer low encapsulation efficiencies, poor control over release, and incomplete shielding of the payloads from environmental stresses (*e.g.*, light, heat, and oxygen).^1,7^ Porous inorganic hosts such as mesoporous silica particles^53^ and vaterite (CaCO_3_)^54,55^ have therefore been explored as alternative carriers, where these offer high specific surface areas and adsorption capacities, and high chemical and thermal stability.^53,56,57^ However, larger payloads often cannot infiltrate small pores,^58^ and the open structure of the host can lead to undesirable leaching. The latter has been addressed using a “gatekeeping” strategy, where the loaded porous hosts are sealed with pore capping agents,^57,59^ but this relies on rigorous design of the porous hosts and complex multi-step synthesis.

Encapsulation within inorganic shells overcomes problems associated with the size of the payload.^12,60–63^ However, these structures are commonly prepared using sacrificial templates, whose removal often necessitates harsh conditions such as elevated calcination temperatures, chemical etching or the use of organic solvents.^61,62,64^ Hollow structures can also be generated as Pickering emulsions, where these are stabilized by solid particles adsorbed at the oil/water interface.^14–16,22^ Although easy to synthesize, the encapsulated cargo invariably leaks through the pores between the particles forming the shell because of the inevitable presence of defects in the particle packing on the droplet surface.^65^ An additional “locking step” is therefore required to create a robust shell, where this can be performed by sintering the system, chemical cross-linking, coating with layers of polymers or depositing a metal layer around the capsules.^22,66,67^ Release of the cargo then requires degradation of the capsule, resulting in an uncontrolled burst release.^68^

As a strategy for achieving complete protection of active molecules, they can instead be incorporated within a dense mineral phase. Amorphous particles such as amorphous calcium carbonate (ACC)^19,69^ and amorphous calcium phosphate (ACP)^70^ have been widely explored due to advantageous properties including high solubilities and biocompatibility. However, they are also inherently unstable, and slowly crystallize under ambient conditions, resulting in short shelf-lives. Encapsulation within single crystals therefore provides an exciting alternative, where the host is stable and provides an effective physical barrier that shields the payloads.^71,72^ For this purpose, considerable effort has been made to unravel the mechanisms by which additives become incorporated within calcite single crystals.^24,29,73–75^ These studies have demonstrated that occlusion is governed by the binding affinity of the additive to the surface of the growing crystal. Efficient occlusion is achieved when an additive binds strongly enough to binding sites on the crystal surface (*i.e.*, kinks and step edges) such that the residence time on the crystal surface exceeds the time taken for steps to propagate around the particles, but not so strongly that crystal growth is inhibited.^74–76^

Uniform incorporation throughout the crystal host thus depends on the additive structure and crystal growth conditions. Efficient incorporation of polymer-stabilized nanoparticles requires judicious design of the additive surface chemistry, where precise control over the chemical functionality, length and density of the steric stabilizers can lead to optimum binding to the crystal surface under a specific crystal growth condition.^34,76^ This can be a tedious process that is specific to each guest species. For example, entrapment of inorganic nanoparticles is only achieved if they are functionalized by macromolecules that can (i) tightly anchor to the particle, (ii) provide the nanoparticle with sufficient colloidal stability in the crystal growth solution and (iii) strongly adsorb to the crystal surface. Further, uniform occlusion is often only achieved at relatively low supersaturations.

These design rules have been employed to create bespoke polymer vesicles that can be entrapped within calcite.^33,34^ However, occlusion was achieved at very low supersaturation levels (*i.e.*, typically [Ca^2+^] ≈ 1.5 mM), limiting the yield of the composite crystals and thus the scalability of this approach. Higher supersaturations generate polycrystalline structures that do not afford complete protection of the payload. Most importantly, molecular species are only entrapped at low levels in these vesicles due to the poor binding affinity between the cargos and the vesicles, and the highly permeable vesicle membrane precludes their use to effectively protect and deliver cargos.^6,33,35,36^ Consequently, no release studies of this calcite/vesicle system have been conducted.

Occlusion of loaded nanogels within calcite crystals therefore offers a significant step-change in methodology and provides the opportunity to finally exploit this unique encapsulation strategy in protection, storage and delivery applications. It offers many advantages. (1) Nanogels are well-established as delivery systems with associated high loading efficiencies.^7–9^ (2) Nanogels containing bioactive molecules can be directly occluded within single crystals of calcite using a facile one-pot method, giving both high loadings and high yields of the composite crystals. (3) The dense mineral completely prevents leakage of the payloads from the carriers, and (4) protects the active species from harsh external conditions (high temperature and pH), thus significantly improving the shelf-life of the payloads. (5) Calcite shows a pH-dependent solubility,^44^ such that a sustained release of the cargos is achieved when the mineral is exposed to an acidic medium, but no leaching occurs at physiological conditions.

It is also instructive to compare this nanogel/calcite hybrid with ACC and vaterite as carriers for active compounds. Both ACC and vaterite have been widely explored as carrier systems, where active compounds are incorporated within the mineral by simple coprecipitation, or in the case of vaterite, by infiltration of actives in the porous structure of the crystals.^77–79^ This is experimentally straightforward but offers much lower loading efficiencies than our nanogel-based approach. Further, both ACC and vaterite have limited shelf-lives due to their instability against crystallization, but can be readily formed with submicron sizes, facilitating their use as intracellular delivery vehicles. However, release/leaching is often observed at physiological conditions – very rapidly in the case of ACC – due to the high solubility of ACC and recrystallization of vaterite. Each mineral host therefore offers distinct features, where the calcite single crystals employed here are unique in their ability to provide an impermeable environment that is ideal for long-term storage and protection of active compounds.

## Conclusions

We have demonstrated that BSA nanogels can be effectively incorporated within calcite single crystals at levels of up to 45 vol% using a simple one-pot method. Exploring DOX and lysozyme as active species, high loading efficiencies within the nanogels were achieved, and their subsequent occlusion in calcite completely prevents uncontrolled leaching, protects the payloads from aggressive external stresses, and enables on-demand controlled release. These micron-scale hybrid crystals can find immediate applications in extending the shelf-life of orally-administered drugs, and in storing and transporting bioactive molecules in challenging environments. It is envisaged that our approach can be extended to the encapsulation and delivery of alternative bioactive compounds, where calcium carbonate alone has low cytotoxicity.^80,81^ This will require nanoscale host calcite crystals, which can be prepared using established methods,^82^ and will form the basis of a future study. Our straightforward and versatile approach therefore offers a significant advance on previous work encapsulating polymer vesicles in calcite crystals as delivery vehicles, where the latter are notoriously leaky and require custom-made copolymers. It also brings new insight into the formulation of payload-carrier conjugates and offers exciting new opportunities to efficiently encapsulate, store and trigger the release of a wide range of guest species.

## Author contributions

O. N. led the experimental work, the synthesis of the samples and carried out the characterization and analysis of the data. A. N. K. performed the FIB-SEM and TGA analyses. T. K. and M. J. D. carried out the solid-state NMR analyses. O. G. G. performed the HRPXRD analyses. Y. Y. K. and O. J. C. helped with the results. O. N. and F. C. M. wrote the manuscript with the contributions from all authors.

## Conflicts of interest

The authors declare no competing financial interest.

## Supplementary Material

SC-012-D1SC02991F-s001
