# Supplementary material for: Incorporation of nanogels within calcite single crystals for the storage, protection and controlled release of active compounds
Source: Chem Sci. 2021 Jun 28;12(28):9839–50. doi: 10.1039/d1sc02991f (PMC8293999; doi:10.1039/d1sc02991f)
Supplement: SC-012-D1SC02991F-s001 [file SC-012-D1SC02991F-s001.pdf]

Electronic Supplementary Material (ESI) for Chemical Science.
This journal is © The Royal Society of Chemistry 2021

**Supplementary Information**

# Incorporation of nanogels within calcite crystals for the storage, protection and controlled release of active compounds

*Ouassef Nahi, Alexander N. Kulak, Thomas Kress, Yi-Yeoun Kim, Ola G. Grendal, Melinda J. Duer, Olivier J. Cayre, and Fiona C. Meldrum*

## 1. Materials

### 1.1. Synthesis of the protein nanogels, loading and incorporation in calcite

Bovine serum albumin (BSA, MW 66.4 KDa), lysozyme from chicken egg white (MW 14.5 kDa), glutaraldehyde (> 99%), doxorubicin hydrochloride (99%), IR820 (cyanine dye, 80%), sodium chloride (NaCl), calcium chloride dihydrate ($CaCl_2.2H_2O$), sodium carbonate ($Na_2CO_3$), ammonium carbonate ($(NH_4)_2CO_3$), sodium hypochlorite (NaClO, 10-15%), and lysozyme assay kit were purchased from Sigma-Aldrich (U.K.) and were used without further purification.

### 1.2. Synthesis of the polymeric vesicles

Benzyl methacrylate (BzMA, 96%) and methacrylic acid (MAA, 99%) monomers were used for the synthesis of poly(methacrylic acid)-poly(benzyl methacrylate) diblock copolymer. The radical polymerisation was initiated by 4,4′-azobis-4-cyanopentanoic acid (ACVA, 98%). 4-cyano-4-(phenylcarbonothioylthio)pentanoic acid was employed as the chain-transfer agent (CTA) of the RAFT polymerisation. Trimethylsilyldiazomethane was used for the methylation of the methacrylic acid residues for the GPC analyses and deuterated dimethyl sulfoxide ($C_2D_6OS$) and chloroform ($CDCl_3$) were used for the $^1$H NMR analyses of the macro-CTA and the block copolymers.

## 2. Synthesis and loading of the BSA nanogels

### 2.1. Synthesis of the BSA nanogels

BSA nanogels were prepared using a previously described protocol.[1] Briefly, 500 mg of BSA proteins were dispersed in 20 mL of deionized water containing 10 mM of NaCl. Under constant stirring (500 rpm), 30 mL of ethanol were slowly added to the mixture at room temperature *via* aid of a syringe pump operating at a flow rate of 1 mL $min^{-1}$. Upon slow addition of ethanol, the solution became milky white, indicating the successful formation of the nanogels. Following the desolvation process, 10 μL of glutaraldehyde (final concentration $10^{-3}$ wt%) were added to the mixture and left overnight under stirring (500 rpm) to complete the cross-linking of the nanogels.

### 2.2. Loading of the BSA nanogels

Desired amounts (0.1 mg $mL^{-1}$ to 1 mg $mL^{-1}$) of payloads (doxorubicin hydrochloride, lysozyme and IR820) were dissolved in 10 mL of deionized water. 1 mL of the BSA nanogels (10 mg $mL^{-1}$) was added dropwise to the solution containing the payloads under constant stirring (500 rpm). The solutions were sonicated for 15 min to aid the encapsulation of the payloads inside the nanocarriers. After 12 hours, the samples were purified with deionized water by repeated centrifugation (7,500 rpm, 10 min). The samples were subsequently redispersed in 10 mL of deionized water.

## 3. Synthesis of the poly(methacrylic acid)-poly(benzyl methacrylate) vesicles

### 3.1. Synthesis of the poly(methacrylic acid) (PMAA) macro-CTA agent

A round-bottomed flask was charged with MAA (5.00 g; 58 mmol), 4-cyano-4-(phenylcarbonothioylthio)pentanoic CTA (279 mg, 0.998 mmol), ACVA (50.0 mg; 0.179 mmol;

[CTA]/[ACVA] = 5.6) and ethanol (5.00 g). The reaction mixture was sealed and purged with nitrogen gas for 30 min and then placed in an oil bath heated at 70 °C for 3 h. The obtained PMAA macro-CTA ($M_n \approx 13{,}500$ g mol$^{-1}$, $M_w \approx 14{,}700$ g mol$^{-1}$, $M_w/M_n \approx 1.09$) was purified by dialysis, first against 1:1 water/methanol and then against pure deionized water. The PMAA macro-RAFT agent was subsequently isolated by lyophilization. A mean degree of polymerisation (DP) of 70 ($PMAA_{70}$) was estimated using $^1$H NMR spectroscopy performed in $CDCl_3$ by comparison of the integrated signal intensity of the aromatic protons of the CTA structure at 7.2-7.4 ppm with that of the methacrylic polymer backbone protons at 0.4-2.5 ppm.

### 3.2. Synthesis of the poly(methacrylic acid)-poly(benzyl methacrylate) block copolymer

BzMA (2.00 g; 11.40 mmol), $PMAA_{70}$ macro-CTA (170 mg; 0.030 mmol) and ACVA (1.60 mg; 0.006 mmol; [macro-CTA]/[ACVA] = 5.00) were charged in a round-bottomed flask containing ethanol (1.871 g). The sealed reaction mixture was purged with nitrogen gas for 30 min and then placed in an oil bath heated at 70 °C for 24 hours. The $^1$H NMR spectroscopy performed in $C_2D_6OS$ showed full conversion of the benzyl methacrylate monomer and a mean DP of PBzMA block of 210 was calculated. GPC analysis performed in THF eluent showed a near-monodisperse molecular weight distribution (Mn ≈ 35,800 g mol$^{-1}$, Mw ≈ 42,600 g mol$^{-1}$, Mw/Mn ≈ 1.19).

### 3.3. Loading of DOX and IR820 in poly(methacrylic acid)-poly(benzyl methacrylate) block copolymer vesicles

Desired amounts (0.1 mg $mL^{-1}$ to 1 mg $mL^{-1}$) of payloads (DOX and IR820) were dissolved in 10 mL of deionized water. 1 mL of the PMAA-PBzMA vesicles (10 mg $mL^{-1}$) was added dropwise to the solution containing the payloads under constant stirring (500 rpm). The solutions were sonicated for 15 min to aid the encapsulation of the payloads inside the nanocarriers. After 12 hours, the samples were purified with deionized water by repeated centrifugation (7,500 rpm, 10 min). The samples were subsequently redispersed in 10 mL of deionized water.

## 4. Encapsulation and release of the (bio)active compounds

### 4.1. Encapsulation measurements

The encapsulation and the loading efficiencies of DOX, lysozyme and IR820 within the BSA nanogels and the vesicles were calculated using the following formulas:

$$\text{Encapsulation Efficiency (weight \%)} = 100 \times \frac{M_T - M_F}{M_T} \quad (1)$$

$$\text{Loading Efficiency (weight \%)} = 100 \times \frac{M_T - M_F}{M_P} \quad (2)$$

where $M_T$ is the total mass of (bio)active compounds fed into the solution, $M_F$ is the mass of free (bio)active compounds that are not encapsulated within the nanoparticles and $M_P$ is the mass of the BSA nanogels/vesicles added to the solution.

### 4.2. Occlusion of the loaded nanocarriers in calcite single crystals and mesoporous vaterite polycrystals

Calcite crystals were precipitated in the presence the loaded protein nanogels and polymeric vesicles using the ammonium diffusion method.[2] Glass substrates were cleaned by soaking in

Piranha solution ($H_2SO_4$ : $H_2O_2$ – 70 vol% : 30 vol%) prior to use. The clean substrates were deposited at the bottom of a well-plate containing 1 mL of solution. 0.50 wt% of the loaded nanogels were added to an aqueous solution containing 20 mM of $CaCl_2$, while the loaded vesicles were mixed with 1.5 mM of $CaCl_2$. Precipitations of the composite crystals were carried out by placing the reaction mixtures inside a sealed desiccator (19 L), containing 2 g of $(NH_4)_2CO_3$, placed in a Petri-dish covered with Parafilm that was punctured several times with a needle. Experiments were carried out at room temperature for 24 h. After the reaction completion, the substrates supporting the crystals were washed several times with deionized water and then ethanol, followed by drying in air prior to characterization.

Incorporation of the loaded nanogels in mesoporous vaterite crystals was carried out using a similar protocol as described above, but in the presence of 1 M of $NH_4Cl$ in the mineralization solution. The presence of ammonium ions in the solution favors precipitation of vaterite polycrystals.[3, 4]

### 4.3. Encapsulation of DOX in amorphous calcium carbonate

Encapsulation of DOX in amorphous calcium carbonate (ACC) was carried out using a coprecipitation method in which a drug feed of 0.5 mg $mL^{-1}$ was mixed with an equimolar mixture (100 mM) of $CaCl_2$ and $Na_2CO_3$. After immediate precipitation, the loaded nanoparticles were isolated by centrifugation (10,000 rpm, 1 min). For Transmission Electron Microscopy (TEM) characterization, the DOX/ACC product was isolated by centrifugation (10,000 rpm, 1 min) and redispersed in ethanol to prevent dissolution of the amorphous particles in aqueous solution.[5]

### 4.4. Release measurements

The payloads-nanogels and the payloads-vesicles were placed in dialysis bags (MWCO = 12 – 14 kDa, SpectraPor). The dialysis bags were then immersed in 50 mL solutions containing the various buffers (PBS or citrate buffers). The samples were dialyzed at physiological temperature of 37 °C with slow stirring (200 rpm). 1 mL aliquots from the releasing phase were collected at various time intervals and were subsequently replaced by the same volume of buffer solution.

Reference solutions of concentrations ranging from 0 mg $mL^{-1}$ to 1 mg $mL^{-1}$ of the various payloads were prepared in buffered solutions to construct the calibration curves using a NanoDrop Microvolume UV/Vis spectrophotometer. The characteristic absorbance peaks of the various active species were monitored over time and compared to the calibration curves to determine the amount of payload encapsulated within the nanogels, the polymer vesicles and the ACC nanoparticles. The release profiles of DOX from the BSA nanogels and the BSA nanogel/calcite composites were determined at physiological pH = 7.4 (PBS buffer; 37 °C) and endosomal/lysosomal pH = 3.5 and 5.5 (citrate buffers; 37 °C). The release profiles of DOX from ACC and the BSA nanogel/vaterite hybrid crystals were carried out at physiological pH = 7.4 (PBS buffer; 37 °C).

## 5. Activity assay of lysozyme

Enzymatic activity measurements of the native and encapsulated lysozyme were carried out using a lysozyme detection kit (Sigma Aldrich, U.K.), according to manufacturer's instructions. Briefly, the lysozyme assay is based on the lysis of *Micrococcus lysodeikticus cells* (substrate),

which results in a decrease of the absorbance at 450 nm over time induced by the change in turbidity of the suspension.

800 µL of *Micrococcus lysodeikticus cells* (0.01 wt%) were dispersed in the reaction buffer (50 mM PBS buffer, pH 6.2) and the solution was then equilibrated to 25 °C, prior to measurements. The final cell suspension had an initial absorbance at $\lambda$ = 450 nm of 0.65 versus the reaction buffer blank. Subsequently 30 µL of the native or encapsulated lysozyme (250 units/mL) dispersed in the reaction buffer was added to the reaction solution. The decrease of absorbance at $\lambda$ = 450 nm was then monitored for 15 min to determine the native and encapsulated lysozyme activities. Untreated native lysozyme was used as control experiment, where the specific activity of the enzyme corresponds to 100%. All activity measurements were repeated twice.

## 6. Characterization of the protein nanogels and the PMAA-PBzMA vesicles

### 6.1. $^1$H NMR Spectroscopy

$^1$H NMR spectra were acquired in deuterated chloroform ($CDCl_3$) and dimethyl sulfoxide ($C_2D_6OS$) solvents, using a Bruker 400 MHz spectrometer.

### 6.2. $^1$H Solid-State NMR Spectroscopy

Solid-state magic angle spinning (MAS) NMR spectroscopy experiments were performed at room temperature on a Bruker AVANCE 400 MHz (9.4 T) instrument equipped with a 4 mm HX probe operating at a spinning rate of 10 kHz. Proton spectra were acquired with a rotor-synchronized Hahn echo (π/2)x-τ-(π)y-τ-acquire that removed proton background signals. The experiment used rf field strength of ca. 67 kHz, an echo time of 100 µs, and a 5 sec recycle

delay. Chemical shifts were referenced externally using residual the water line of neat $D_2O$ at 4.79 ppm. Powders of the BSA nanogel/calcite hybrid heated at 100 °C for 2 h (heating ramp = 10 °C $min^{-1}$) were analyzed and compared to the non-heated BSA nanogel/calcite composite crystals and pure calcite single crystals.

### 6.3. Gel Permeation Chromatography (GPC)

The GPC set-up comprised an Agilent 1260 Infinity system fitted with two 5 µm Mixed-C columns and a guard column, a refractive index detector and an UV/Vis detector operating at 309 nm. PMAA homopolymer and PMAA-PBzMA copolymer were dissolved in THF eluent containing 2.0 vol% triethylamine and 0.050 vol% butylhydroxytoluene (BHT). A series of near-monodisperse poly(methyl methacrylate) standards were employed for the calibration, using the refractive index and the UV/Vis detectors for determining the molecular weights and molar mass dispersities of the standards.

For the GPC analyses, the methacrylic acid residues present in both the PMAA macro-CTA and the PMAA-PBzMA block copolymer were methylated using trimethylsilyldiazomethane to prevent the adsorption of the polymer onto the GPC column.[6] Briefly, the $PMAA_{70}$ macro-CTA and the $PMAA_{70}$-$PBzMA_{210}$ block copolymer were first dissolved in THF and then trimethylsilyldiazomethane was added dropwise at room temperature. On addition, the solution became yellow and bubbles appeared. After a few seconds, the solution became colorless and a few more drops of trimethylsilyldiazomethane were added until the yellow color of the solution withstood, and the solution stopped bubbling. The solution was then stirred overnight at room temperature.

### 6.4. Dynamic Light Scattering (DLS)

The hydrodynamic diameters and size distributions by intensity of the protein nanogels and polymer vesicles were measured using Dynamic Light Scattering (DLS). The measurements were carried out using a Malvern Zetasizer NanoZS apparatus at a fixed scattering angle of 173°. Aqueous solutions of the BSA nanogels and the $PMAA_{70}$-$PBzMA_{210}$ vesicles (0.10 wt%) were prepared for the analyses (Figure S1).

### 6.5. Electron Microscopy

Samples for Transmission Electron Microscopy (TEM) were prepared by placing a 15 µL droplet of an aqueous or ethanolic suspension of 0.10 wt% nanoparticles on a TEM grid for 1 min. Excess solution was removed *via* blotting. Copper TEM grids coated with a carbon film were employed, and these were treated with a plasma glow discharge for 30 sec to create a hydrophilic surface prior to addition of the aqueous nanogels dispersion. TEM was conducted using a FEI Tecnai TF20 FEGTEM with an Oxford Instruments INCA 350 EDX system/80 mm X-Max SDD detector and a Gatan Orius CCD camera operating at 200 kV.

Scanning Electron Microscopy (SEM) was carried out using a FEI NanoSEM Nova 450 to image the BSA nanogels and the PMAA-PBzMA vesicles. The samples were mounted on SEM stubs using carbon adhesive discs and were coated with a 4 nm iridium layer to enable imaging of the non-conductive organic nanoparticles. To image the incorporation of the nanoparticles occluded within the calcite host crystals, sample milling was performed using FEI Helio G4 CX dual beam-high resolution monochromated FEG SEM instrument equipped with a Ga Focused

Ion Beam (FIB). A selected area of the crystals was pre-coated with 2 μm thick Pt. The operating voltage was 30 kV and the Ga-ion currents were varied between 2.5 nA and 7 nA.

### 6.6. Single-Crystal XRD

Measurements of the hybrid crystals were carried out at 130 K using an Agilent SuperNova diffractometer equipped with an Atlas CCD detector and connected to an Oxford Cryostream low-temperature device using mirror monochromated Mo Kα radiation (λ = 0.71073 Å) from a Microfocus X-ray source. Diffraction patterns were processed using CrysAlisPro software.

### 6.7. Synchrotron High-Resolution PXRD

Pure calcite single crystals and calcite incorporating high levels of DOX-loaded BSA nanogels were analyzed using synchrotron high-resolution powder X-ray diffraction (HR-PXRD) on beamline ID22 at the European Synchrotron Research Facility (ESRF), Grenoble, France, at a wavelength of 0.35449587 ± 0.00000467 Å. Instrument calibration was carried out using a high purity Si(111) standard. The instrumental contribution to the peak broadening does not exceed 0.003° (2θ), and peak positions and shapes are accurate and reproducible to a few tenths of a millidegree. The powder samples were loaded into 0.5 mm borosilicate glass capillaries and the diffractograms were recorded at room temperature (Figure S3).

### 6.8. Thermogravimetric measurements

Thermogravimetric analyses (TGA) were performed using a TA-Instruments Q600 SDT operating at 10 °C $min^{-1}$ from 20 °C to 850 °C in air. The samples were bleached in NaClO 10-15% solution for 6 h prior to characterization to remove the surface bound organic matter.[7]

Calcination of the pure calcite reference shows an onset of decomposition at 650 °C, giving a weight loss of 44.0 wt% that is ascribed to the release of $CO_2$(g), leaving a residue of 56.0 wt% that corresponds to CaO(s) (Figure S4). Thermal decomposition of the BSA nanogels indicates that 16.5 wt% of the nanogels remain as ashes, even after annealing at 850 °C. Pyrolysis of the composite crystals indicates a high level of incorporation of the BSA nanogels within calcite, where 14.7 wt% of organic decomposition is detected between 200 °C and 600 °C. Further, assuming that all calcite crystals decompose at 850 °C into $CO_2$(g) + CO(s), then an excess of 8.5 wt% remains in the crucible alongside the CO(s) residues. This most likely corresponds to the remaining BSA nanogels that do not fully decompose at 850 °C. Overall, this equates to ≈ 23.2 wt% of BSA nanogels incorporated within calcite crystals and corresponds to approximately 45 vol% of the composite materials, based on the BSA nanogels density of 1 g $cm^{-3}$ (given high water contents).

### 6.9. Circular Dichroism (CD)

Circular Dichroism (CD) spectroscopic measurements were carried out using a Chirascan Plus (Applied Photophysics) spectropolarimeter equipped with a Peltier system for controlling the temperature. The CD spectra of the native and encapsulated lysozyme (200 µg $mL^{-1}$) were recorded in the far UV region (185 nm – 260 nm) using 1 nm increments with a bandwidth of 2 nm and 1 sec time per point. Samples were pre-equilibrated at 20 °C prior to analysis and each measurement was repeated twice. The secondary structure of lysozyme was obtained by subtracting the spectra of the appropriate blank media without lysozyme from the recorded enzyme spectra. To measure the melting point (Tm) value of the untreated native lysozyme, the buffered solution containing the enzyme was heated from 5 °C to 90 °C and

then cooled down from 90 °C to 5 °C, and the evolution of the characteristic absorbance band at 209 nm was monitored (Figure S6).

### 6.10. Other measurements

Optical micrographs of the loaded BSA nanogels and PMAA-PBzMA vesicles occluded within calcite single crystals were recorded using a Nikon Eclipse LV100 polarizing microscope, equipped with both transmitted and reflected light sources. Fluorescence microscopy images of the BSA nanogels loaded with doxorubicin were recorded using a Zeiss Axio Scope A1 microscope fitted with an AxioCam monochrome camera light source. The Raman spectra of the BSA nanogels incorporated within the calcite and vaterite host crystals were recorded with a Renishaw 2000 Raman Microscope equipped with a 785 nm diode laser (Figure S2). Fourier transform infrared spectra (FTIR) were acquired over the mid infrared region (from 600 $cm^{-1}$ to 4000 $cm^{-1}$) using sing a Perkin–Elmer ATR-IR instrument (Figure S5 and Figure S8). The surface area and pore size analyses of the mesoporous vaterite crystals were measured using $N_2$(g) absorption with a Micromeritics-TriStar 3000 after degassing for 2 h the crystals at 100 °C (Figure S9).

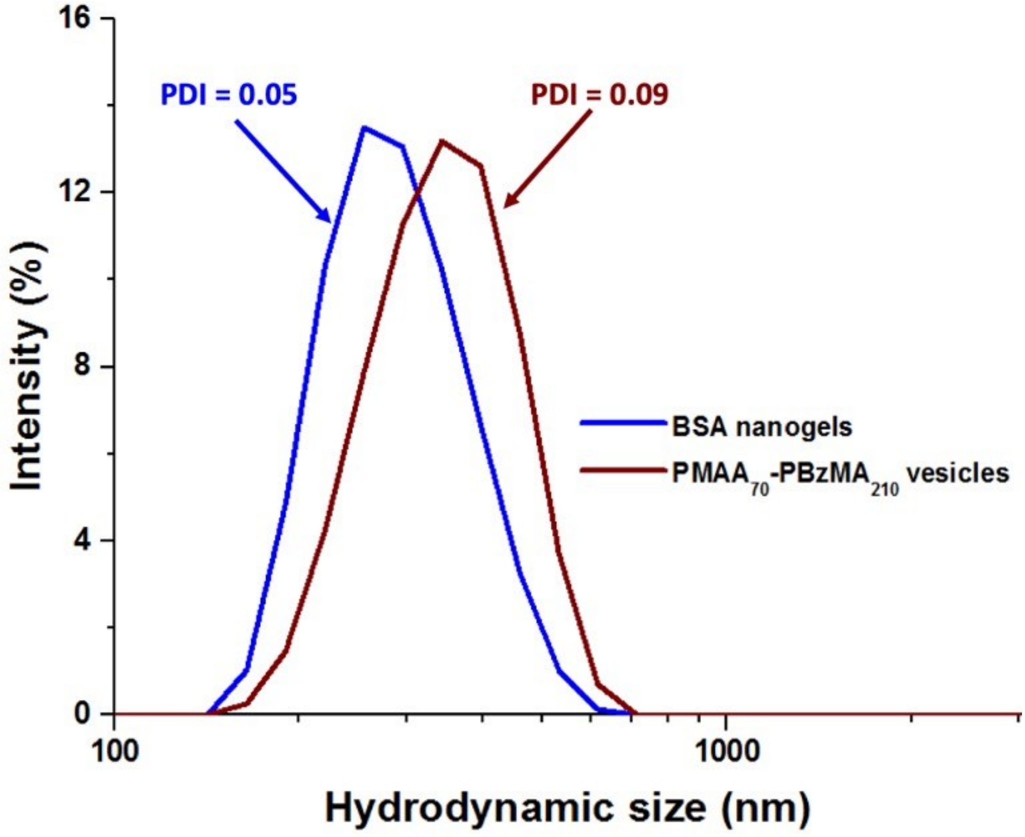


**Figure S1.** Size distribution by intensity obtained by Dynamic Light Scattering (DLS) of the BSA nanogels and $PMAA_{70}$-$PBzMA_{210}$ vesicles (0.10 wt%) in aqueous medium. Both nanoparticles have unimodal and narrow size distributions, as indicated by their low polydispersity indices (PDI).

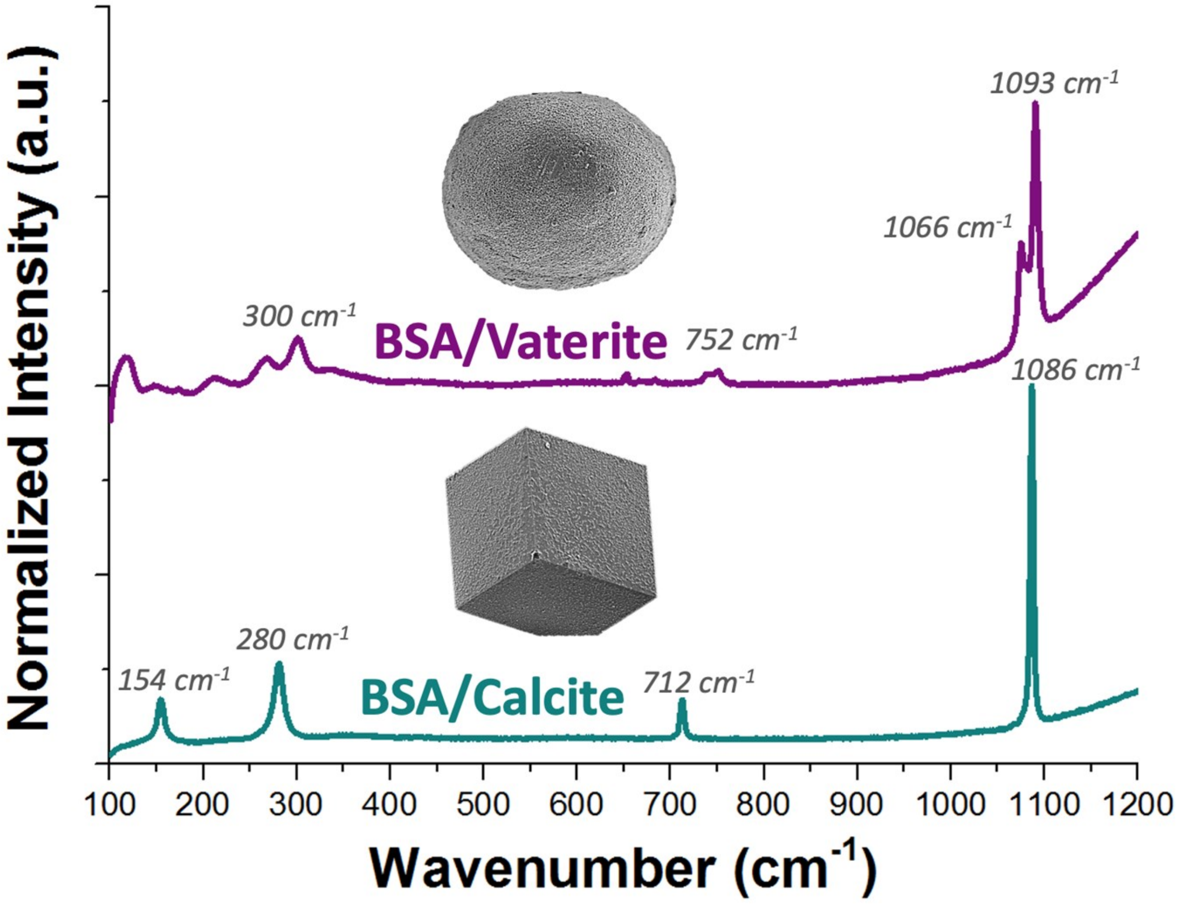


**Figure S2.** Raman spectra of the BSA nanogel-$CaCO_3$ composite crystals, confirming the polymorph of the host crystals, where the characteristic $\nu_1$ (1086 $cm^{-1}$), $\nu_4$ (712 $cm^{-1}$) and lattice modes (154 $cm^{-1}$ and 280 $cm^{-1}$) of calcite (blue) and $\nu_1$ (1093 $cm^{-1}$ and 1066 $cm^{-1}$), $\nu_4$ (752 $cm^{-1}$) and lattice mode (300 $cm^{-1}$) of vaterite (purple) are recorded.

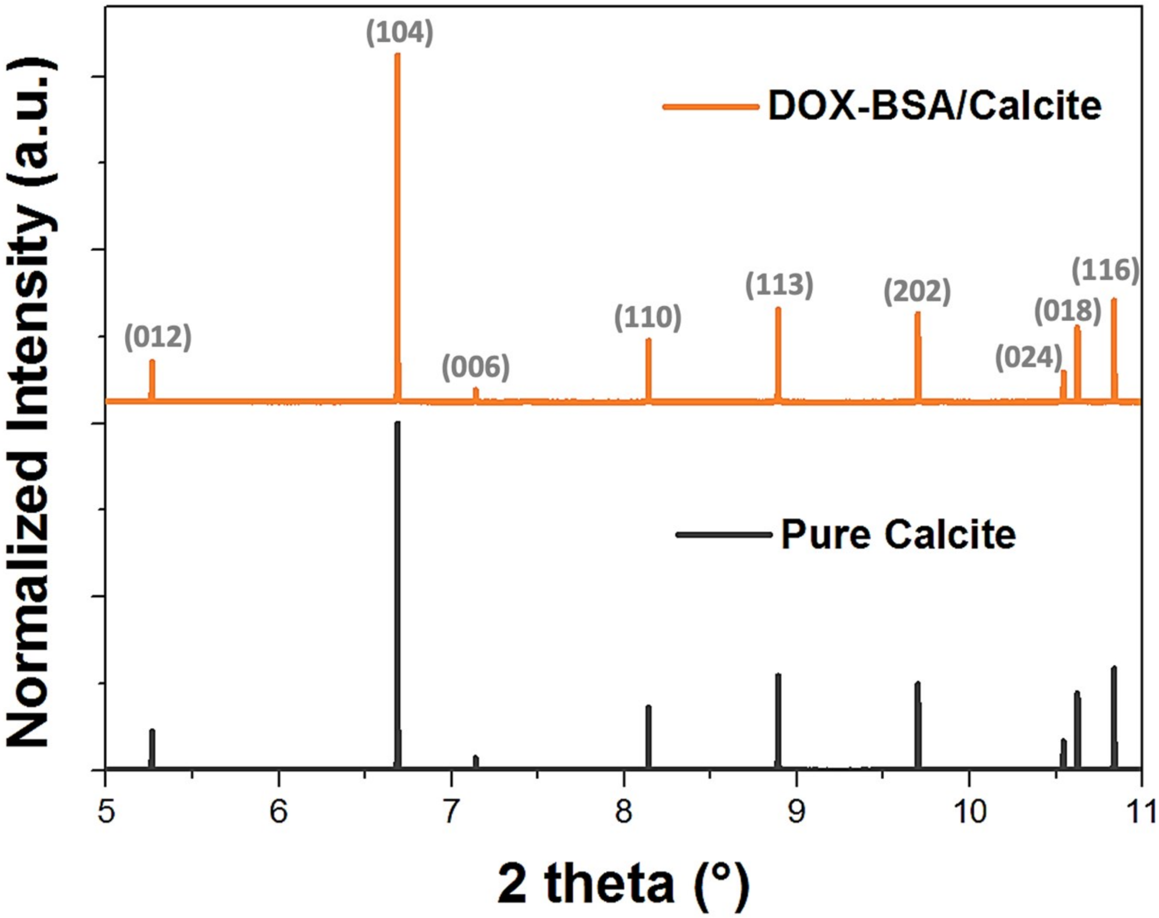


**Figure S3.** HR-PXRD diffractograms of the DOX-BSA nanogel-$CaCO_3$ composite materials, confirming the polymorph of the host crystals, where the characteristic diffraction peaks of calcite are recorded.

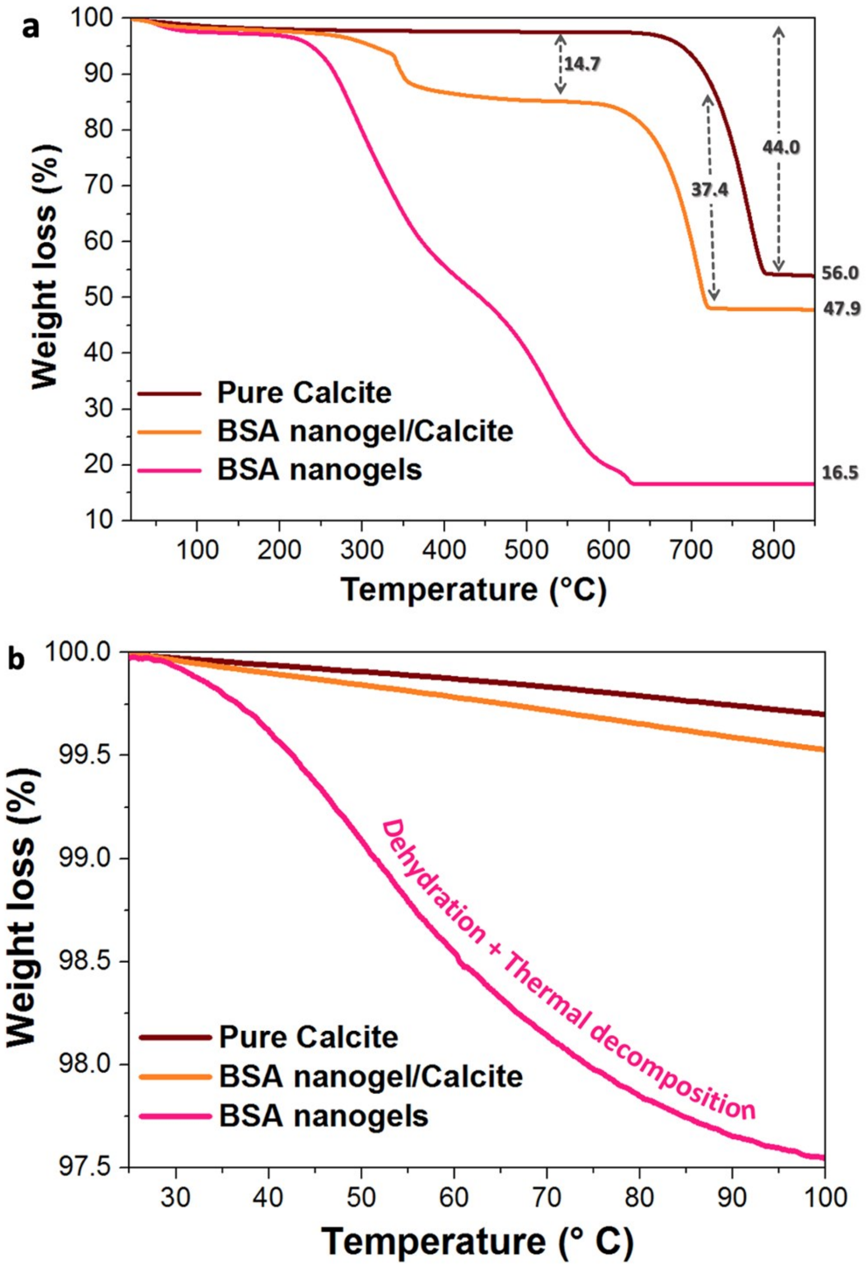


**Figure S4.** (a) Thermogravimetric analyses (TGA) recorded in air for the pure calcite reference (dark red), the free BSA nanogels (pink) and the BSA nanogels occluded within calcite single crystals (orange). (b) Focus on the region between 25 °C and 100 °C of the thermograph showing that, conversely to the free BSA nanogels, no dehydration or thermal decomposition is recorded for the BSA nanogel/calcite system. Note that the 0.25 wt% weight loss observed for the BSA/calcite crystals compared to the pure calcite crystals is very little compared to that of the free BSA nanogels (2.5 wt%) and within the instrument accuracy range.

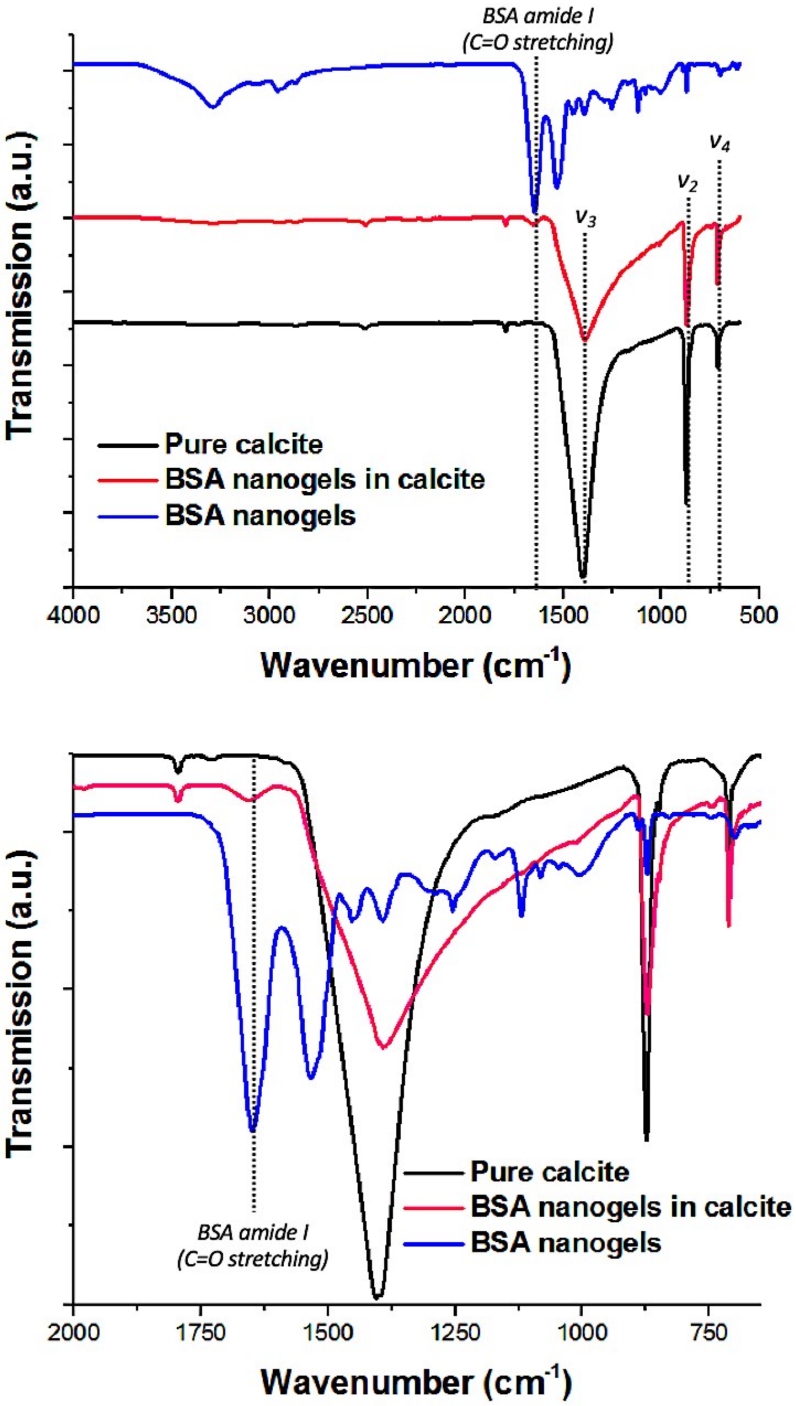


**Figure S5.** FTIR spectra of pure calcite crystals (black), calcite crystals occluding the BSA nanogels (pink) and freeze dried BSA nanogels (blue). The characteristic peaks of calcite $\nu_2$ (out-of-plane $CO_3$ bending), $\nu_3$ (in-plane asymmetric $CO_3$ stretching) and $\nu_4$ (in-plane $CO_3$ bending) and the presence of the main BSA peak (1650 $cm^{-1}$, amide I corresponding to C=O stretching) show that the BSA nanogels are occluded in the calcite host crystals.

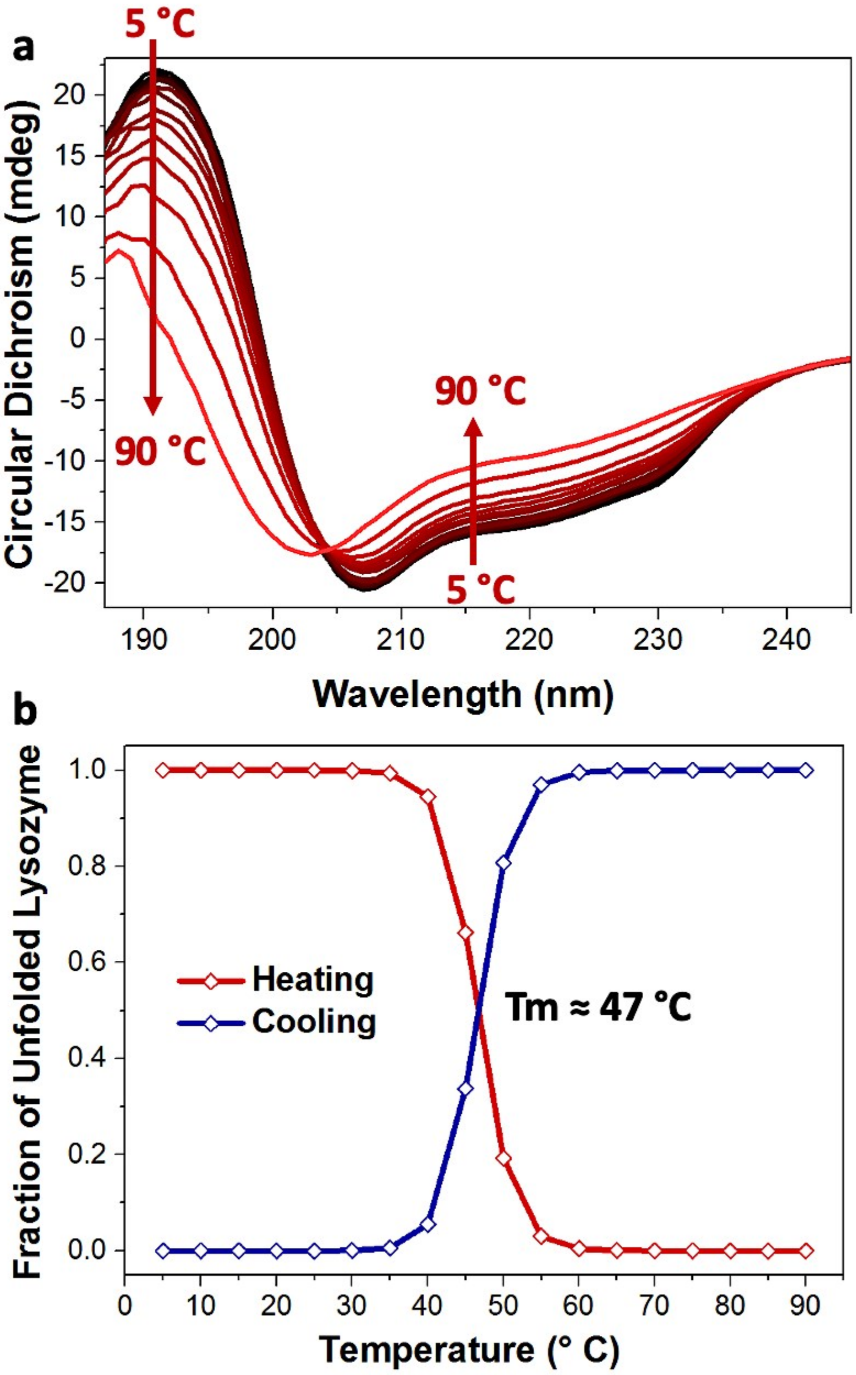


**Figure S6.** (a) Temperature-dependent evolution of the CD spectra of native lysozyme showing a decrease of intensity with increasing temperature of the characteristic peaks of the enzyme. Note that the peak at 209 nm is also shifted to 203 nm when increasing the temperature. (b) Normalized fraction of unfolded/folded lysozyme extracted from temperature-dependent evolution of the CD spectra of the native enzyme. These results demonstrate instability/denaturation of the native lysozyme when the temperature is increased.

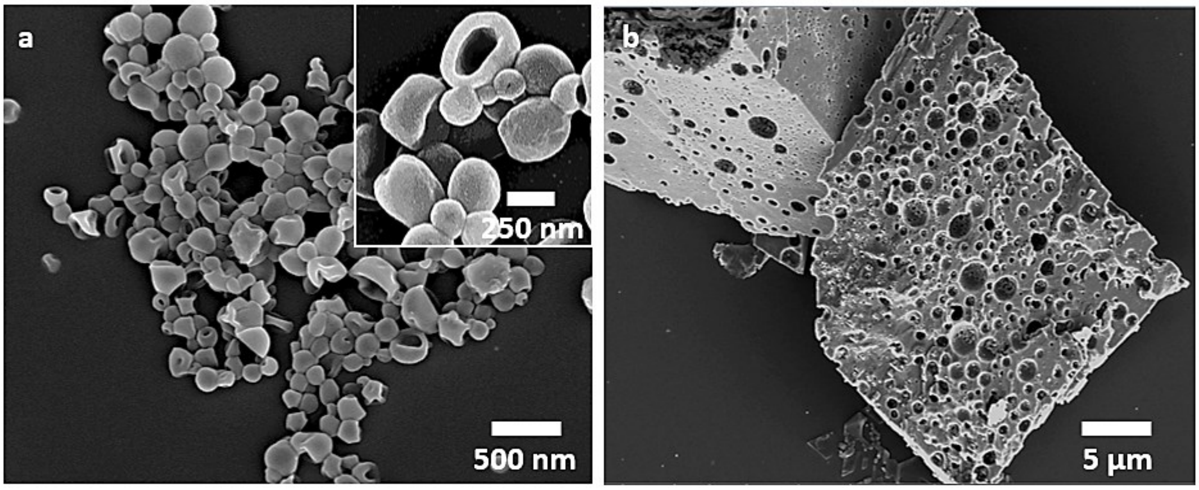


**Figure S7.** Scanning Electron Micrographs of the dry $PMAA_{70}$-$PBzMA_{210}$ vesicles (a) and a fractured crystal showing the efficient occlusion of the vesicles throughout calcite (b).

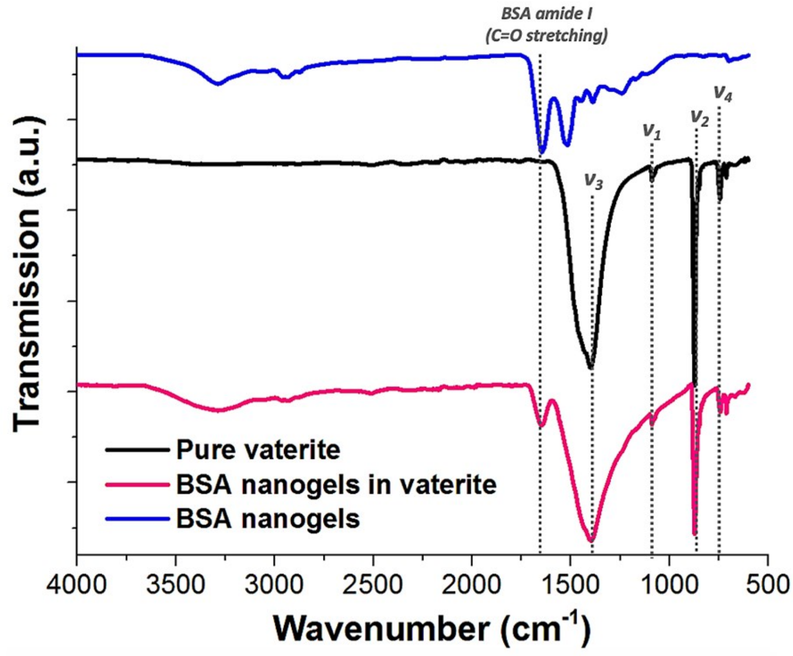


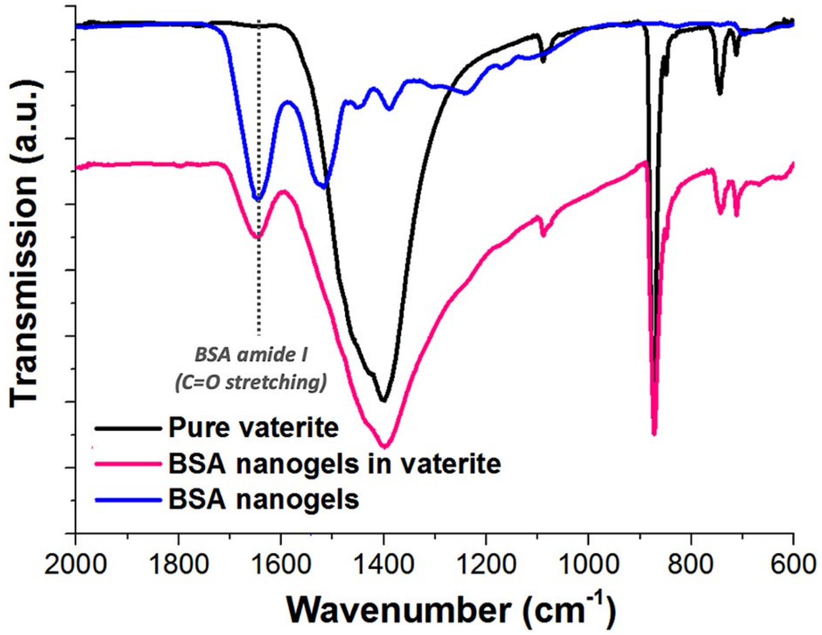


**Figure S8.** FTIR spectra of pure vaterite crystals (black), calcite crystals occluding the BSA nanogels (pink) and freeze dried BSA nanogels (blue). The characteristic peaks of vaterite $\nu_1$ (symmetric $CO_3$ stretching), $\nu_2$ (out-of-plane $CO_3$ bending), $\nu_3$ (in-plane asymmetric $CO_3$ stretching) and $\nu_4$ (in-plane $CO_3$ bending) and the presence of the main BSA peak (1650 $cm^{-1}$, amide I corresponding to C=O stretching) show that the BSA nanogels are occluded in the vaterite host crystals.

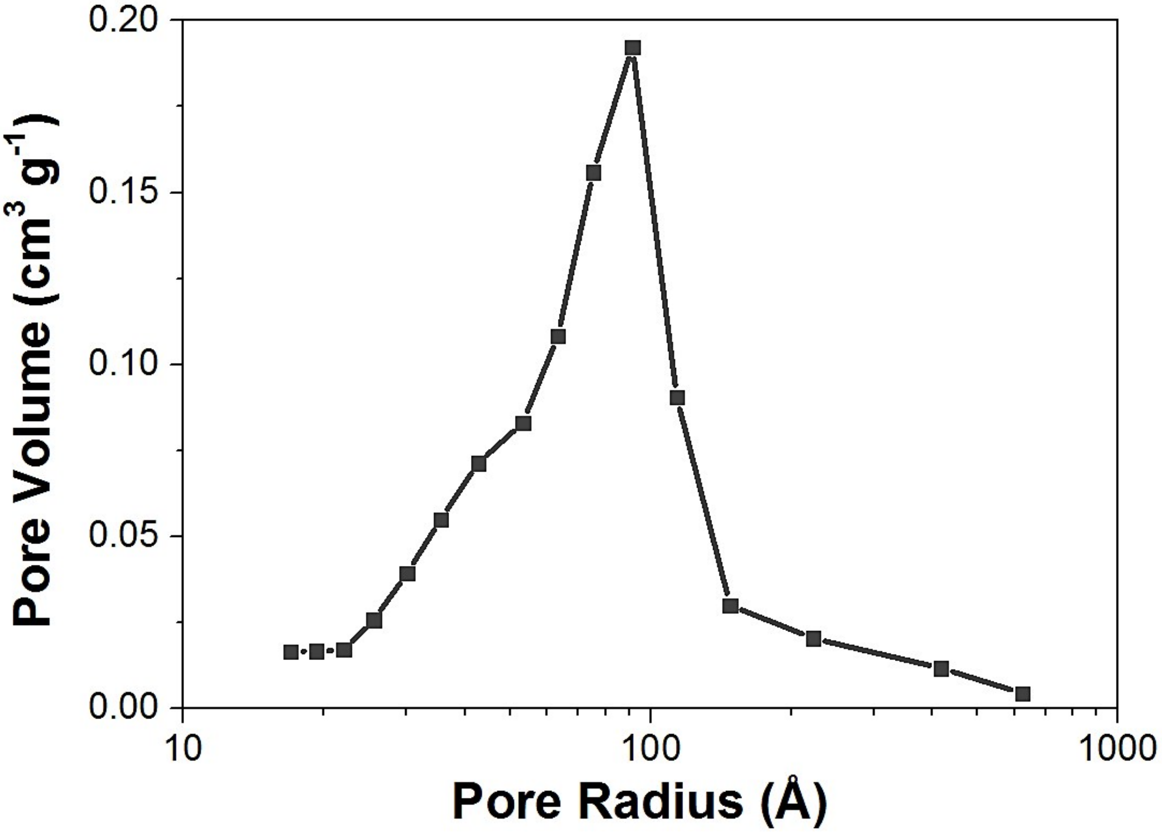


**Figure S9.** Pore size distribution of the DOX-BSA nanogel/vaterite polycrystals revealed by Brunauer-Emmett-Teller (BET) analysis.

**REFERENCES**

1. Z. Yu, M. Yu, Z. B. Zhang, G. Hong and Q. Q. Xiong, *Nanoscale Res. Lett.*, 2014, **9**, 343-350.
2. J. Ihli, P. Bots, A. Kulak, L. G. Benning and F. C. Meldrum, *Adv. Func. Mater.*, 2013, **23**, 1965-1973.
3. N. Gehrke, H. Cölfen, N. Pinna, M. Antonietti and N. Nassif, *Cryst. Growth Des.*, 2005, **5**, 1317-1319.
4. E. M. Pouget, P. H. H. Bomans, A. Dey, P. M. Frederik, G. de With and N. A. J. M. Sommerdijk, *J. Am. Chem. Soc.*, 2010, **132**, 11560-11565.
5. S.-F. Chen, H. Cölfen, M. Antonietti and S.-H. Yu, *Chem. Commun.*, 2013, **49**, 9564-9566.
6. A. A. Cockram, T. J. Neal, M. J. Derry, O. O. Mykhaylyk, N. S. J. Williams, M. W. Murray, S. N. Emmett and S. P. Armes, *Macromolecules*, 2017, **50**, 796-802.
7. K. E. H. Penkman, D. S. Kaufman, D. Maddy and M. J. Collins, *Quat. Geochronol.*, 2008, **3**, 2-25.
